# Research on High-Precision Measurement Technology of the Extinction Ratio Based on the Transparent Element Mueller Matrix

**DOI:** 10.3390/mi16070781

**Published:** 2025-06-30

**Authors:** Ruiqi Xu, Mingpeng Hu, Xuedong Cao, Jiahui Ren

**Affiliations:** 1 National Key Laboratory of Optical Field Manipulation Science and Technology, Chinese Academy of Sciences, Chengdu 610209, China; 19908251078@163.com (R.X.); cxd@ioe.ac.cn (X.C.); 18834167036@163.com (J.R.); 2 Institute of Optics and Electronics, Chinese Academy of Sciences, Chengdu 610209, China; 3 University of Chinese Academy of Sciences, Beijing 100049, China

**Keywords:** transmissive optical component, extinction ratio, polarization measurement, stokes vector

## Abstract

With the widespread application of optical technology in numerous fields, the polarization performance of transmissive optical components has become increasingly crucial. The extinction ratio, an important indicator for evaluating their polarization characteristics, holds great significance for its precise detection. Aiming at the measurement of the extinction ratio of a transparent component, this study proposes a measurement method for solving the extinction ratio based on measuring the Mueller matrix of the transparent component. The purpose is to analyze the worst position of the extinction ratio of the transmissive component. The extinction ratio of the sample is obtained according to the phase retardation derived from the Stokes vector of the incident light and the Mueller matrix of the optical component, and a theoretical analysis and simulation of this method are carried out. The simulation results verify the feasibility of the theoretical derivation of this method. To further verify the accuracy of the measurement method, experimental verification is conducted. A standard transparent sample with a phase retardation of 13 nm is selected for actual measurement. The data of independent experiments on the transparent sample under different powers are analyzed, and the extinction ratio of the transparent sample is further obtained. When using this method, the relative error is less than 2%, indicating good accuracy.

## 1. Introduction

In the modern field of optics [1,2,3,4], transparent optical components, such as polarizers and wave plates, are widely used in numerous cutting-edge technologies, including optical communication, optical imaging, laser processing, and quantum optics [5,6,7,8]. The precise manipulation of its polarization performance directly affects the stability and work efficiency of the whole optical system in signal transmission, image acquisition, material processing, and quantum state preparation. The extinction ratio, a key parameter for evaluating the selective transmission ability of a transparent component to different polarization states, not only affects the quality grading and performance calibration of optical components but also determines the overall performance of optical systems [9]. In optical communication systems, accurate detection of the extinction ratio can significantly enhance the signal-to-noise ratio, ensuring the stability and reliability of data transmission [10]. In biomedical imaging, a high extinction ratio improves image contrast and clarity, making the analysis of polarization characteristics in biological tissues more precise, which aids in achieving higher-precision image analysis and processing, thereby enhancing the ability to diagnose diseases early [11,12]. In quantum optics experiments, the accurate measurement of the extinction ratio is essential for achieving quantum information transmission and processing [13].

Currently, there are various methods for detecting the extinction ratio of a transparent component [14,15,16,17,18]. Traditional methods based on mechanical rotating parts work by changing the polarization direction through mechanical rotation of polarization elements (such as polarizers) to achieve intensity modulation. Typically, a detector measurement method is used, where the light source, polarizer, the element to be tested, analyzer, and detector are arranged in sequence along the optical path. The light emitted by the light source is converted into linearly polarized light by the polarizer and then passes through the measured element and the analyzer, and the intensity is measured by the detector. During one full rotation of the mechanical component, the maximum and minimum readings of the detector are recorded, and the extinction ratio is calculated. The detection process requires the polarization device to rotate through 360 degrees to obtain the extinction ratio, which is not only time-consuming and inefficient but also difficult to meet the industrial batch detection requirements. Moreover, it is affected by mechanical vibration and other factors, which restricts its application in high-precision optical element detection [19,20]. The heterodyne interferometry-based detection method uses a laser as the system light source. The input light is converted into linearly polarized light by a polarizer and then split into two beams by a polarizing beam splitter. One beam undergoes mechanical delay, while the other undergoes frequency shift and polarization adjustment. The two beams are incident on the *X*-axis and *Y*-axis of a polarization-maintaining fiber (PMF), respectively. During the transmission of polarized light, there is a coupling effect. By adjusting the delay distance, only one polarized beam participates in the interference. The extinction ratio is calculated based on the detection of the spatial distribution of the coupling. Although the detection accuracy of this method is high and breaks through the accuracy bottleneck of traditional technology, the employment of heterodyne interferometry for extinction ratio measurement introduces frequency shifts and entails relatively complex operational procedures, thereby presenting challenges of high technical costs in practical engineering applications [21,22,23]. The technical limitations of the aforementioned detection methods fundamentally reflect the inherent trade-offs in optical detection when pursuing high precision and efficiency simultaneously. These limitations not only impede the quality enhancement and performance optimization of transparent optical components but also impose constraints on multiple technological frontiers: the expansion of transmission capacity in optical communication networks, the breakthroughs in resolution for optical imaging systems, the precision improvement of laser processing equipment, and the reliability assurance in quantum optics experiments. Therefore, developing a high-precision, efficient, and cost-effective method for detecting the extinction ratio of a transparent component is not only a critical research direction in the current field of optical detection technology but also a key driver for advancing the optical field towards higher performance and greater intelligence.

In response to the above research situation, this work proposes a new method for detecting the extinction ratio of a transparent component. Firstly, the theoretical analysis of the worst extinction ratio value and its position of the transparent sample is conducted. Secondly, the formulas for calculating the phase retardation and stress direction of the transparent sample optical component are derived using the Stokes vector [24,25] and Mueller matrix [26,27,28], and the extinction ratio of the sample is calculated based on the phase retardation of the transparent sample optical component [29]. In the experiment, the data of independent experiments on the transparent sample are analyzed. The experimental process is simple, with good repeatability and high accuracy. The final experimental results also verify the effectiveness and reliability of this method.

## 2. Principle Analysis

### 2.1. Derivation of Extinction Ratio

**Figure 1:**
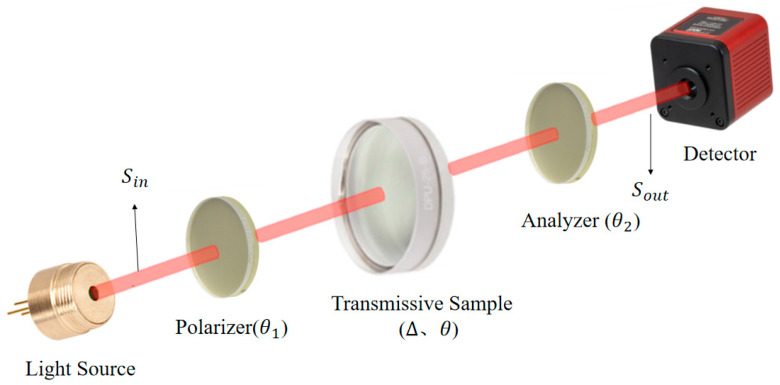
Schematic diagram of the transmission path for measuring the extinction ratio of a transparent component.

The schematic diagram of the transmission optical path is shown in Figure 1 [30]. The light source starts along the *z*-axis and becomes linearly polarized after passing through a polarizer, with its transmission axis in the y-direction. It then vertically incidents on the surface of the sample under test. After transmission, the polarization state of the outgoing light changes, and finally, the light intensity signal is received by the detector after passing through an analyzer [31].

The extinction ratio of the transmissive element is measured. In the transmissive model, the light source is modulated into linearly polarized light by the polarizer and then passes through the transmissive element and the analyzer, and finally, the detector detects the polarization state of the beam [32,33].

Its system model is shown in the following formula [34]:

(1) $$\;S_{out}=M_{q}MM_{j}S_{in}$$

Among them, $S_{in}\;$ and $S_{out}\;$ denote the Stokes vectors of the incident light and the beam detected by the detector. The Stokes vectors of the monochromatic light source $S_{in}\;$ with an intensity of $I_{0}\;$ is as follows:

(2) $$\;S_{in}=\left[\begin{array}{c} I_{0}\\ 0\\ 0\\ 0 \end{array}\right]$$

$\;M_{j}\;$ and ${\;M}_{q}\;$ represent the Mueller matrices of the polarizer and analyzer, whose expressions are shown as follows [35,36,37]:

(3) $$\;M_{j}{=M}_{q}=\frac{1}{2}\left[\begin{array}{cccc} 1 & cos2\theta & sin2\theta & 0\\ cos2\theta & {cos}^{2}2\theta & sin2\theta cos2\theta & 0\\ sin2\theta & sin2\theta cos2\theta & {sin}^{2}2\theta & 0\\ 0 & 0 & 0 & 0 \end{array}\right]$$

The parameter M is the Mueller matrix of the transparent component, which is expressed as follows:

(4) $$\;M=\left[\begin{array}{cccc} 1 & 0 & 0 & 0\\ 0 & {cos}^{2}2\theta +{sin}^{2}2\theta cos\Delta & (1-cos\Delta )sin2\theta cos2\theta & -sin2\theta sin\Delta \\ 0 & (1-cos\Delta )sin2\theta cos2\theta & {sin}^{2}2\theta +{cos}^{2}2\theta cos\Delta & cos2\theta sin\Delta \\ 0 & sin2\theta sin\Delta & -cos2\theta sin\Delta & cos\Delta \end{array}\right]$$

As can be seen from the expression, the transmissive element contains two parameters: the angle θ with respect to the *x*-axis (where the fast axis is θ) and the retardation ∆.

According to the definition of the Stokes vector, the first parameter $S_{0}$ of the Stokes vector represents the total light intensity [38,39]. Therefore, the expression for the light intensity of the beam emerging from the polarizer can be derived as follows:

(5) $$I=\frac{1}{4}\left(1+\cos2{(\theta }_{2}-\theta_{1}\right)-2{sin}^{2}\frac{\Delta }{2}\sin\left(\theta -\theta_{1}\right)sin2(\theta -\theta_{2}))I_{0}$$

Among them, $\theta_{1}$ and $\theta_{2}$ are the angles between the polarizer and analyzer with the *x*-axis direction, respectively.

Let $x=\theta_{2}-\theta_{1}$; then, Equation (5) can be further rearranged into a function of x:

(6) $$I\propto 1+\sqrt{A^{2}+B^{2}}\cos\left(2x+\delta \right)$$

Among which

(7) $$A=1-2{sin}^{2}\frac{\Delta }{2}\sin\left(\theta -\theta_{1}\right)sin2(\theta -\theta_{1})$$

(8) $$B=2{sin}^{2}\frac{\Delta }{2}\sin\left(\theta -\theta_{1}\right)sin2(\theta -\theta_{1})$$

The maximum light intensity is $1+\sqrt{A^{2}+B^{2}}$, and the minimum light intensity is $1-\sqrt{A^{2}+B^{2}}$; thus, the extinction ratio is as follows:

(9) $$PER=\frac{1+\sqrt{A^{2}+B^{2}}}{1-\sqrt{A^{2}+B^{2}}}$$

When $\sqrt{A^{2}+B^{2}}$ is minimized, the extinction ratio is at its worst.

(10) $$A^{2}+B^{2}=1-8{sin}^{2}\frac{\Delta }{2}{sin}^{2}ycosy+4{sin}^{2}\frac{\Delta }{2}{sin}^{2}y$$

Here, $y=\theta -\theta_{1}$. Through analysis, it is found that, when $y=\pm \frac{\pi }{4}$, the influence of the cross term $8{sin}^{2}\frac{\Delta }{2}{sin}^{2}ycosy$ is greatest, resulting in the minimum value of $A^{2}+B^{2}$.

Theoretical derivation from the expression shows that, when the polarizer angle $\theta_{1}$ differs from the fast-axis angle θ of the transmission device by $\pm \frac{\pi }{4}$, i.e., $\theta_{1}=\theta \pm \frac{\pi }{4}$, at this point, the extinction ratio reaches its worst value, with the worst value being

(11) $$PER=\cot^{2}\frac{∆}{2}$$

### 2.2. Analysis of Transmission Device Parameters

The polarizer at an angle $\theta_{2}$ to the *x*-axis is divided into four polarization directions, 0°, 45°, 90°, and 135°, which can be expressed as follows:

(12) $$P_{i}\frac{1}{2}\left[\begin{array}{cccc} 1 & cos2(\theta_{2}+k_{i}) & sin2(\theta_{2}+k_{i}) & 0\\ cos2(\theta_{2}+k_{i}) & {cos}^{2}2(\theta_{2}+k_{i}) & sin2(\theta_{2}+k_{i})cos2(\theta_{2}+k_{i}) & 0\\ sin2(\theta_{2}+k_{i}) & sin2(\theta_{2}+k_{i})cos2(\theta_{2}+k_{i}) & {sin}^{2}2(\theta_{2}+k_{i}) & 0\\ 0 & 0 & 0 & 0 \end{array}\right]$$

When i=1,2,3,4, $k_{i}=0{}^{\circ},45{}^{\circ},90{}^{\circ},135{}^{\circ}$.

Then, the Stokes vector of the outgoing light after passing through the polarizer can be derived as follows:

(13) $$I_{i}=P_{i}MM_{j}S_{in},\;(i=1,2,3,4;)$$

According to Formula (5), taking $\theta_{1}=0$ and $\theta_{2}=0$, we obtain

(14) $$I_{1}=\frac{I_{0}}{2}-\frac{I_{0}}{2}{sin}^{2}\frac{\Delta }{2}{sin}^{2}(2\theta )$$

Similarly, it can be obtained that $I_{2}$, $I_{3}$, and $I_{4}$ are

(15) $$I_{2}=\frac{I_{0}}{4}+\frac{I_{0}}{2}{sin}^{2}\frac{\Delta }{2}sin(2\theta )cos(2\theta )$$

(16) $$I_{3}=\frac{I_{0}}{2}{sin}^{2}\frac{\Delta }{2}{sin}^{2}(2\theta )$$

(17) $$I_{4}=\frac{I_{0}}{4}-\frac{I_{0}}{2}{sin}^{2}\frac{\Delta }{2}sin(2\theta )cos(2\theta )$$

It can be deduced that

(18) $$\Delta =2arcsin\sqrt{\frac{{\left(2I_{3}\right)}^{2}+{\left(I_{2}-I_{4}\right)}^{2}}{4I_{3}(I_{1}+I_{3})}}$$

(19) $$\theta =\frac{1}{2}arctan(\frac{I_{3}-I_{1}+I_{2}{+I}_{4}}{I_{2}-I_{4}})$$

It can be observed that, when solving the parameters of transparent samples—retardation Δ and fast axis α—they are only related to the intensity of light.

Based on the above, this paper proposes a new method: by fixing the polarizer at 0° and utilizing the light intensity values obtained from rotating the analyzer to four angles (0°, 45°, 90°, 135°), the sample information of the transmission-type device can be calculated, thereby determining the extinction ratio of the sample, which is represented by the following equation:

(20) $$PER=\cot^{2}\frac{∆}{2}=\cot^{2}\left(arcsin\sqrt{\frac{{\left(2I_{3}\right)}^{2}+{\left(I_{2}-I_{4}\right)}^{2}}{4I_{3}(I_{1}+I_{3})}}\right)$$

## 3. Simulation Results and Analysis

As can be theoretically derived from the above, the specific relationship between the total intensity of the outgoing light and the total intensity of the incident light is as follows:

(21) $$I=\frac{1}{4}\left(1+\cos2{(\theta }_{2}-\theta_{1}\right)-2{sin}^{2}\frac{\Delta }{2}\sin\left(\theta -\theta_{1}\right)sin2(\theta -\theta_{2}))I_{0}$$

Among them, $\theta_{1}$ and $\theta_{2}$ are the angles between the polarizer and analyzer with the *x*-axis direction, respectively.

**Figure 2:**
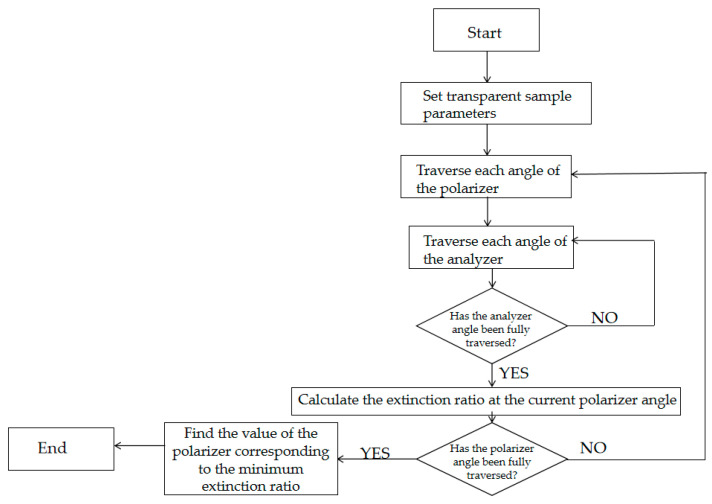
System logic framework diagram for measuring the extinction ratio based on the traditional method of mechanical rotating parts.

In order to verify the feasibility of the theoretical derivation of the method in this paper, the traditional method simulation was carried out through Matlab R2024b, and the extinction ratio obtained after simulation was compared with the extinction ratio obtained by this method. The simulation flow chart is shown below in Figure 2.

**Figure 3:**
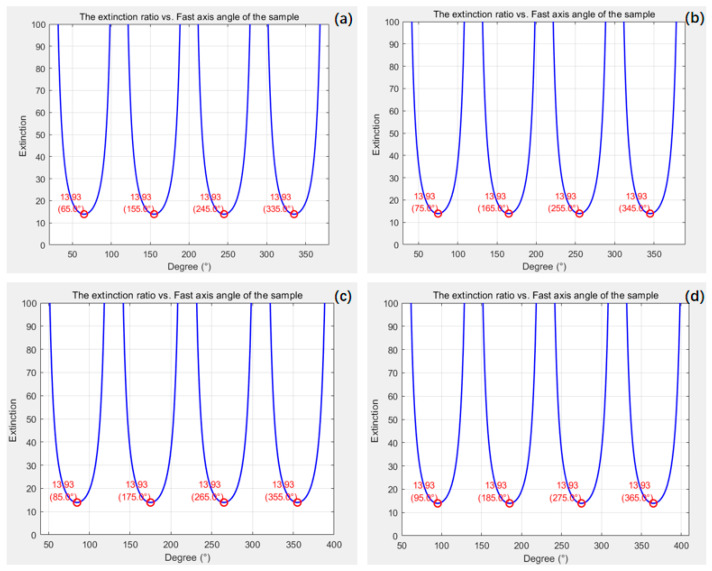
The relationship curve between polarizer angle and fast axis angle of the transparent sample when measuring the extinction ratio: (**a**) fast axis angles = 20°; (**b**) fast axis angles = 30°; (**c**) fast axis angles = 40°; (**d**) fast axis angles = 50°.

Set Δ = 30 nm and the fast axis angles to 20°, 30°, 40°, and 50°, respectively. It can be verified that, when the angle between the polarizer and the fast axis direction differs by $\frac{\pi }{4}$, the extinction ratio is at its worst. The specific relationship curve and corresponding table are shown in Figure 3 and Table 1.

As shown in Figure 2, the PER obtained from traditional simulation methods is 13.93. When Δ=30 nm is set, $PER=\cot^{2}\frac{∆}{2}=13.93$, which can be calculated by the method proposed in this paper. Through simulation experiments, the feasibility of the theoretical derivation of this method has been verified.

## 4. Experiments and Results Analysis

**Figure 4:**
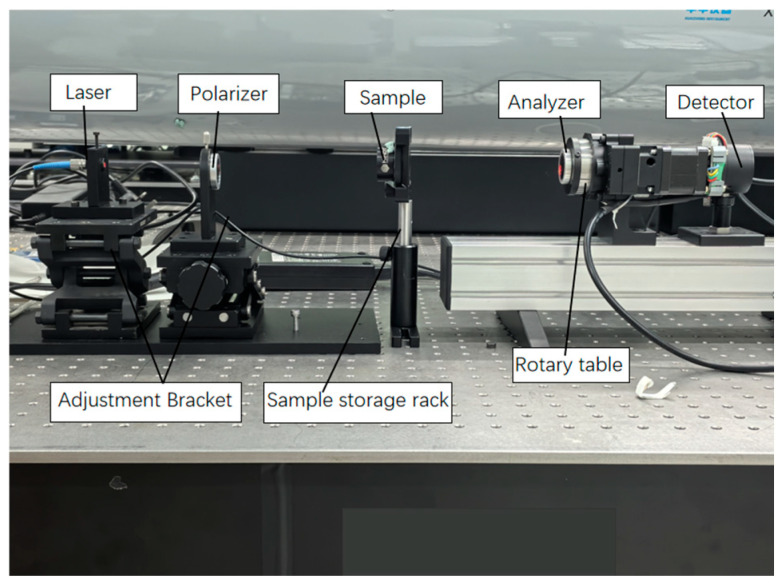
Schematic diagram of the experimental device for measuring the extinction ratio of transparent elements.

With reference to the aforementioned optical path, an experimental platform was constructed, and an experimental system was designed as shown in Figure 4. The system employs a laser with a wavelength of 633 nm as the visible light source, a linear polarizer with a spectral range of 400 nm to 700 nm, and an extinction ratio > 10,000:1 as the polarizer. The analyzer is mounted on a rotary stage, with the rotation angle controlled by the stage, and the final outgoing light is received by a detector. In this experiment, a standard stress 13 nm retardation plate produced by Hinds Instruments was selected for the actual measurement.

**Figure 5:**
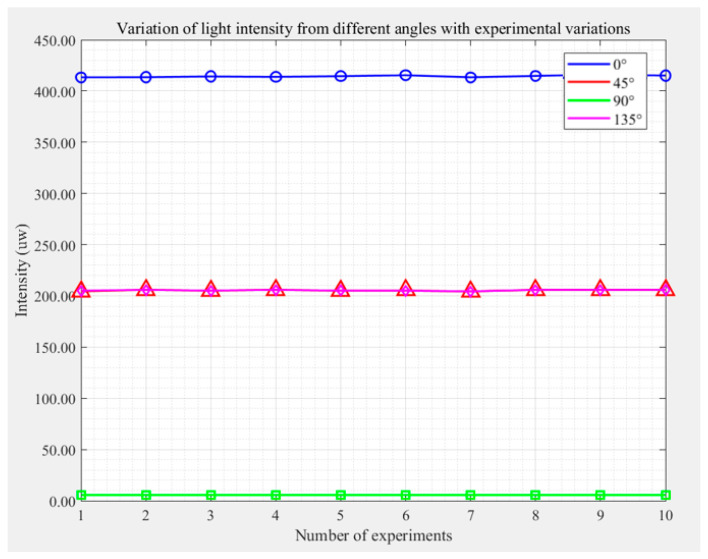
The variation curve of the light intensity received by the detector under different angles (0°, 45°, 90°, 135°) of the polarizer with the number of experiments.

Through analysis, it was found that the detector’s response to light intensity has the greatest impact on the test results. To evaluate its effect on experimental repeatability, this work conducted 10 independent experiments, measuring the light intensity values detected by the probe at different angles (0°, 45°, 90°, 135°). The experimental results are shown in Figure 5 and Table 2. In Table 2, the unit of measured light intensity at different angles is μW, and the unit of retardation of the sample to be tested is nm. Figure 5 shows the values obtained by continuous light intensity detection at different angles of the analyzer. It can be seen that the light intensity fluctuation is very small, and the influence on the experiment can be ignored. Additionally, the data from the 10 experiments in Table 2 were analyzed. By statistically analyzing the mean values (central tendency), standard deviations (dispersion degree), and relative standard deviations (RSD) of each parameter, the stability and reliability of the experimental results were verified. The data analysis results are shown in Table 3.

To analyze whether changes in laser power would affect the experimental results, the laser power was adjusted, and 10 independent experiments were conducted, measuring the light intensity values detected by the detector at different angles. The experimental results are shown in Table 4. In this table, the unit of measured light intensity at different angles is μW, and the unit of retardation of the sample to be tested is nm.

We conducted data analysis on these 10 experiments after power adjustment, verifying the stability and reliability of the experimental results through statistical analysis of parameter means (central tendency), standard deviations (dispersion degree), and relative standard deviations (RSD). The data analysis results are shown in Table 5.

### 4.1. Analysis of Experimental Results

#### 4.1.1. Repetitive Analysis

Repeatability is a crucial metric for evaluating the reliability of experiments, assessed through the relative standard deviation (RSD). In the data from Table 3, the RSD in the i = 1 direction is as low as 0.0022, demonstrating exceptionally high repeatability. The RSD values for the i = 2 and i = 4 directions are 0.0032 and 0.0031, respectively, while the i = 3 direction shows an RSD of 0.0074. Overall, these results indicate minimal fluctuations in light intensity measurement values across multiple experiments, confirming the excellent repeatability of the experimental outcomes.

Similarly, the data in Table 5 exhibit favorable repeatability characteristics, with RSD values of 0.0009, 0.0036, and 0.0050 for the i = 1, i = 2, and i = 4 directions, respectively—closely matching the corresponding RSD values in Table 3, indicating comparable repeatability performance. Although the RSD in the i = 3 direction in both Table 3 and Table 5 is slightly higher, this may stem from minor environmental disturbances (e.g., subtle airflow-induced optical path perturbations) or slight fluctuations in detector sensitivity at the corresponding light intensity levels. Nevertheless, the standard deviations remain at a low level, exerting negligible impact on the overall experimental results and falling within an acceptable range.

These findings demonstrate that the measurement results consistently maintain relative stability across different experimental groups, proving that the experimental system exhibits strong repeatability and provides reliable data support for the experimental conclusions.

#### 4.1.2. Calculate the Extinction Ratio

After averaging the sample retardation Δ measured in 10 different experiments, the result was 13.08 nm. Compared with the actual value of Δ = 13 nm, the relative error was 0.62%, which is relatively small. When Δ = 13 nm, $PER=\cot^{2}\frac{∆}{2}=77.03$. By calculating the extinction ratio using the obtained parameters, the worst-case extinction ratio of the final sample was determined to be 76.09, yielding a relative error of 1.22% compared to the actual value. The small relative error validates the feasibility of this method.

## 5. Discussion

In the field of optical detection, two methods are widely used for the measurement of the extinction ratio: the traditional mechanical rotation detection method and the measurement method based on heterodyne interferometry. While both methods have been extensively employed in both research and industrial applications, they exhibit limitations when compared to the novel methodology proposed in this work, as detailed below.

Traditional detection methods based on mechanical rotation rely on the rotation of polarization elements to modulate light intensity by changing the polarization direction. While this method is simple and cost-effective, it has significant limitations. To obtain maximum and minimum light intensity readings, the polarizer must be rotated 360 degrees to measure the light intensity at each angle, which makes the measurement process time-consuming. Additionally, the mechanical vibrations from the rotating component can introduce errors, reducing measurement accuracy to about 7%. In contrast, the method proposed in this research does not require the polarizer to rotate 360 degrees; instead, it only needs to rotate four angles to complete the extinction ratio measurement, which greatly improves the experimental efficiency. Furthermore, the method demonstrates good repeatability, with negligible impact on the reliability of the overall experimental results across multiple trials.

The measurement method based on heterodyne interferometry uses a laser as the light source. The input linearly polarized light is split into two beams by a polarization beam splitter. One beam undergoes mechanical delay, while the other is frequency-shifted and polarized. The extinction ratio is then calculated based on the spatial distribution of the coupled light after interference in the polarization-maintaining fiber. Although this method can achieve high-precision measurements with an error rate as low as 3%, its complexity is a major drawback. The precise frequency modulation, fiber coupling, and fine adjustment of the delay distance make the experimental setup and operation cumbersome. In contrast, the proposed method employs a non-interferometric technique to measure the parameters in the Mueller matrix of the transparent component to calculate the extinction ratio. This approach simplifies the optical setup, reduces the number of optical components, and eliminates the need for complex frequency modulation operations. Consequently, the proposed method is better suited for practical applications, including on-site and real-time measurements.

To sum up, compared with the traditional mechanical rotation and heterodyne interferometry methods, the method of measuring the extinction ratio based on the Mueller matrix of transparent elements proposed in this work has a precision of less than 2% and shows good performance in terms of measurement accuracy, measurement efficiency, operation simplicity, and adaptability to a variety of measurement scenarios.

## 6. Conclusions

This study proposes a high-precision measurement method for the extinction ratio of a transparent component based on its Mueller matrix. First, a theoretical analysis and derivation were conducted on the extinction ratio and its position in the worst-case scenario for the transparent sample. Second, using Stokes vector and Mueller matrix theory, formulas were derived to calculate the phase delay and stress direction of the transparent component. These formulas, combined with the initial theoretical analysis, ultimately resulted in a formula for calculating the extinction ratio of the transparent component. The proposed method is theoretically simulated and compared with traditional methods, verifying the feasibility of its theoretical derivation. Experimental measurements were conducted on a transparent crystal with a retardation value of Δ = 13 nm. By performing 10 independent repeated tests, the acquired data was analyzed and compared. The results showed a low relative standard deviation (RSD), indicating good experimental reproducibility. The final measured sample retardation Δ exhibited a relative error of 0.62% compared to the theoretical value, indicating minimal deviation. Furthermore, the extinction ratio of the transparent sample was determined, with a relative error of 1.22% compared to the theoretical value, validating the effectiveness of the method. Compared with the existing methods, this method realizes the simplification and efficiency improvement of the device and provides a new idea for the rapid detection of industrial polarization devices. It has the characteristics of high precision, high efficiency, and low cost.

## Figures and Tables

**Table 1:** Relationship table between polarizer angle and fast axis angle.

Fast Axis Angle Degree/°	Polarizer Angle Value at Minimum Extinction Ratio Degree/°	Minimum Extinction Ratio (*PER*)
20	65	13.93
30	75	13.93
40	85	13.93
50	95	13.93

**Table 2:** Independent experimental measurement results.

Number of Experiments	0° Light Intensity μW	45° Light Intensity μW	90° Light Intensity μW	135° Light Intensity μW	The Value of the Sample Parameter Δ Nm	Extinction Ratio
1	413.37	204.30	5.41	204.84	13.07	76.20
2	413.43	205.60	5.50	205.73	13.14	75.39
3	414.18	204.96	5.48	204.87	13.12	75.62
4	413.79	205.65	5.46	205.56	13.11	75.74
5	414.50	205.14	5.50	205.35	13.14	75.39
6	415.41	205.32	5.40	204.85	13.04	76.56
7	413.37	204.20	5.39	204.18	13.01	76.91
8	414.77	205.65	5.44	205.61	13.07	76.20
9	416.01	205.83	5.47	205.71	13.09	75.97
10	415.08	205.87	5.42	205.76	13.03	76.68

**Table 3:** Error analysis table.

Parameter	Mean Value	Standard Deviations	RSD
0°	414.69	0.91	0.0022
45°	205.25	0.66	0.0032
90°	5.44	0.04	0.0074
135°	205.29	0.64	0.0031
Δ	13.09	0.05	0.0038
extinction ratio	76.07	0.54	0.0071

**Table 4:** Independent experimental measurement results after power alteration.

Number of Experiments	0° Light Intensity μW	45° Light Intensity μW	90° Light Intensity μW	135° Ight Intensity μW	The Value of the Sample Parameter Δ Nm	Extinction Ratio
1	258.58	123.45	3.41	123.54	13.10	75.85
2	258.68	124.29	3.35	122.60	13.05	76.44
3	258.18	123.32	3.31	122.63	12.99	77.15
4	258.46	123.09	3.38	122.74	13.07	76.20
5	258.29	123.31	3.40	122.94	13.10	75.85
6	258.56	123.93	3.39	123.67	13.07	76.20
7	258.40	123.96	3.38	123.84	13.05	76.44
8	258.50	123.64	3.40	123.76	13.09	75.97
9	258.89	123.60	3.37	123.64	13.02	76.80
10	258.79	123.61	3.37	123.36	13.03	76.68

**Table 5:** Error analysis table after power change.

Parameter	Mean Value	Standard Deviations	RSD
0°	258.58	0.23	0.0009
45°	123.63	0.45	0.0036
90°	3.38	0.03	0.0089
135°	123.27	0.62	0.0050
Δ	13.06	0.05	0.0038
extinction ratio	76.36	0.63	0.0083

## Data Availability

The original contributions presented in this study are included in the article; further inquiries can be directed to the corresponding author.
